# Effectiveness of 0.66% Povidone-Iodine Eye Drops on Ocular Surface Flora before Cataract Surgery: A Nationwide Microbiological Study

**DOI:** 10.3390/jcm10102198

**Published:** 2021-05-19

**Authors:** Rosario Musumeci, Pasquale Troiano, Marianna Martinelli, Matteo Piovella, Claudio Carbonara, Scipione Rossi, Giovanni Alessio, Luisa Molteni, Claudio Giuseppe Molteni, Laura Saderi, Giovanni Sotgiu, Clementina Elvezia Cocuzza

**Affiliations:** 1 Department of Medicine and Surgery, University of Milano-Bicocca, 20900 Monza, Italy; marianna.martinelli@unimib.it (M.M.); luisa.molteni1295@gmail.com (L.M.); claudio.molteni@unimib.it (C.G.M.); clementina.cocuzza@unimib.it (C.E.C.); 2 MicroMiB Biorepository at the University of Milano-Bicocca, Associated Member of the JRU MIRRI-IT, Via Cadore, 48-20900 Monza, Italy; 3 Department of Ophthalmology, Fatebenefratelli Sacred Family Hospital, 22036 Erba (CO), Italy; ptroiano@fatebenefratelli.eu; 4 Monza Outpatient Microsurgery Center, 20900 Monza, Italy; piovella@piovella.com; 5 Nursing Home Villa Valeria, 00141 Rome, Italy; CARBOEYE1@gmail.com; 6 San Carlo di Nancy Hospital, 00165 Rome, Italy; scipione.rossi@fastwebnet.it; 7 Department of Ophthalmology-Policlinico Hospital of Bari, 70124 Bari, Italy; giovanni.alessio@uniba.it; 8 Clinical Epidemiology and Medical Statistics Unit, Department of Medical, Surgical, Experimental Sciences, University of Sassari, 07100 Sassari, Italy; lsaderi@uniss.it (L.S.); gsotgiu@uniss.it (G.S.)

**Keywords:** povidone iodine, cataract, ocular surgery, endophthalmitis, antiseptic prophylactic treatment

## Abstract

A multicenter, nonrandomized, prospective, controlled study was conducted to evaluate, as perioperative prophylactic treatment, the anti-infective effectiveness of 0.66% povidone-iodine eye drops (IODIM^®^) against the bacterial flora of the conjunctival surface of patients who undergo cataract surgery. Eye drops containing 0.66% povidone-iodine were applied to the eye undergoing cataract surgery; the untreated contralateral eye was used as control. One hundred and twenty patients set to receive unilateral cataract surgery were enrolled in 5 Italian Ophthalmology Centers and pretreated for three days with 0.66% povidone-iodine eye drops. The contralateral eye, used as control, was left untreated. Conjunctival swabs of both eyes were collected at the baseline visit and after three days of treatment, just before the cataract surgery. A qualitative and quantitative microbiological analysis of bacterial presence was evaluated by means of bacterial culture, followed by identification. Methicillin resistance determination was also performed on staphylococci isolates. Bacterial load before and after treatment of the eye candidate for cataract surgery was evaluated and compared to the untreated eye. A reduction or no regrowth on the culture media of the bacterial load was observed in 100% of the study subjects. A great heterogenicity of bacterial species was found. The 0.66% povidone-iodine eye drops, used for three days prior to cataract surgery, were effective in reducing the conjunctival bacterial load. The 0.66% povidone-iodine eye drops (IODIM^®^) might represent a valid perioperative prophylactic antiseptic adjuvant treatment to protect the ocular surface from microbial contamination in preparation of the surgical procedure.

## 1. Introduction

Cataract surgery is a very frequent ophthalmic procedure performed worldwide, and it is considered to be relatively safe [1]. However, endophthalmitis is one of the most serious complications that is often responsible for post-surgical visual impairment [2]. Its incidence is changing over time [3] due to the improvement of surgical techniques and related instrumentation. Most recent data show an incidence of less than 0.01% [4].

Among various risk factors responsible for endophthalmitis are microbial pathogens—numerous Gram-positive and Gram-negative bacteria as well as fungi [5,6,7]—that reflect the aqueous contamination during surgery with bacterial flora from the ocular surface [8]. 

Endophthalmitis prophylaxis varies widely worldwide, and recently it evolved extensively. Preoperative measures included lid hygiene to reduce conjunctival flora, as well as the use of perioperative topical and systemic antibiotics, despite the lack of level 1 evidence confirming their real efficacy. Furthermore, routine intracameral antibiotic prophylaxis is being used increasingly, based on growing observational evidence [9,10].

On the other hand, resistance to antimicrobial agents continues to emerge worldwide, with multidrug-resistant organisms becoming increasingly common [11]. For this reason, the use of antiseptics might represent a valid alternative, allowing the reduction of the overuse or misuse of antibiotics. Antiseptics have a nonselective mechanism of action that often prevents the development of resistance [12]. The European Society of Cataract and Refractive Surgeons guidelines reports that antisepsis with povidone-iodine (PVI) or chlorhexidine (CHX) is mandatory to reduce ocular surface colony counts, prior to cataract surgery [13]. CHX, due to the larger size of its molecule, often cannot penetrate bacterial cell walls and it is also considered much more irritating to the ocular mucosa compared to PVI [12]. Moreover, some studies indicate that the overall exposure to CHX can increase the risk of developing antibiotic resistance, so it seems reasonable to restrict the use of CHX only in cases where there are clear indications for its use [14].

The PVI, developed in the early 1950s, is a chemical compound of polyvinylpyrrolidone and iodine with a rapid broad-spectrum activity against bacteria, fungi, viruses, and protozoa [15]. Povidone matrix acts as a reservoir of “free” iodine, which contributes to the microbicidal activity of PVI solution. As povidone is hydrophilic and has an affinity for cell membranes, it acts as a carrier transferring free iodine (I_2_) directly to the target cell surface, which is the critical event for its microbicidal action [16]. Free iodine iodinates and oxidizes by inactivating key cytosolic proteins, fatty acids, enzymes, and nucleotides, resulting in rapid destruction of the prokaryotic cell [15].

To date, PVI is a standard of care in preoperative prophylaxis for cataract surgery. The 10% PVI solution is applied to the periocular skin, whereas PVI 5% is applied to the cornea and conjunctival sac prior to surgery, as recommended by the American Academy of Ophthalmology and European Society of Refractive Surgeons guideline [13]. However, despite the great advantages of PVI in terms of antisepsis, it is an acidic solution (pH ≤ 5) and might irritate mucous membranes or wounds. PVI 5% solutions were shown to damage the corneal epithelium when placed topically, with an irritant effect related to the duration of exposure. For this reason, 5% PVI cannot be used in the patient ocular surface preparation in the days before surgery. In contrast, the use of low concentrations of PVI, such as 1.0%, 0.5%, and 0.25%, was shown to be well-tolerated, prior to ophthalmic surgery, as reported by Jiang et al. [17]. Moreover, as previously demonstrated by Berkelman et al., diluted PVI solution has greater bactericidal activity than stock solution (10%); probably due to a higher concentration of free iodine in diluted PVI solution [18]. 

Based on these previous findings, efforts were made to evaluate the antimicrobial efficacy of further diluted concentrations of PVI for the pre-surgical application to the ocular surface. 

Isenberg et al. showed that a low concentration of PVI 1.25% or 2.5% solution, applied three times daily in the first postoperative week, reduced the conjunctival bacterial colony-forming units to a similar degree to that of broad-spectrum antibiotic [19]. Similar results were obtained, more recently, by the same author, demonstrating that no significant difference existed between the effect of topical 1.25% PVI solution and commercial topical antibiotics, for treatment of bacterial keratitis [20]. Shimada et al. reported that repeated irrigation of 0.25% povidone-iodine solution achieved an extremely low bacterial contamination rate in the anterior chamber at the completion of cataract surgery [21]. Another in vitro study demonstrated that multiple applications (three every 30-s) of PVI at low concentration (0.7%) was as effective as the use of a bactericidal agent [22].

The bactericidal effect of a new formulation based on 0.66% PVI (IODIM^®^, Medivis) was compared in vitro to that of 5% PVI, against different species of bacteria. The results showed that more diluted 0.66% preparation was more rapidly bactericidal than 5% PVI formulation [23]. More recently, Pinna et al. reported the antimicrobial effect of the same formulation against both bacterial and *Candida* strains, demonstrating an excellent inhibition of growth for bacterial strains after 1 min exposure, and a complete inhibition of *Candida* strains within 24 h [24]. Another clinical study reported the efficacy of 0.6% PVI in reducing conjunctival bacterial load, following needle contamination in patients undergoing anti-VEGF intravitreal injection [25]. 

The aim of this clinical study was to evaluate the bactericidal effect of 0.66% PVI (IODIM^®^, Medivis) administered three times per day, for three days prior to surgery, as an adjuvant perioperative prophylactic treatment in patients undergoing cataract surgery. The 0.66% PVI formulation used in this study was a preservative-free nanoparticellar solution containing hyaluronic acid, medium-chain triglycerides, and low-concentration PVI whose microbicidal efficacy was based on the peculiar ability of the PVI complex low-concentration to release high concentrations of free iodine (I_2_) in water. Therefore, it was necessary to develop a nano-emulsifier that is able to capture I_2_ in water and is able to release the gas after instillation in the conjunctival sac of patients. 

## 2. Materials and Methods

### 2.1. Experimental Design

This is a multi-center, prospective, open-label clinical study that lasted four days, to evaluate the efficacy of 0.66% PVI eye drops against ocular bacterial flora, before cataract surgery.

A total of 120 patients undergoing crystalline extraction, in 5 clinical centers distributed over the national territory, were enrolled in this study. Following the first visit (T_0_), two drops of IODIM^®^ (medical device containing 0.66% PVI, Hyaluronic Acid, Medium Chain Triglycerides, Medivis, Catania, Italy) were applied three times a day to the conjunctival sac of the eye to be operated on, in every subject enrolled in the study, up to the morning of the cataract surgery (T_1_). During the two visits (T_0_ and T_1_), a conjunctival swab collection was performed on each eye (the untreated contralateral eye was used as a control).

### 2.2. Participants 

One hundred and twenty patients, 24 subjects undergoing cataract extraction from each of the 5 participating centers (Erba, Monza, Rome 1 and Rome 2, and Bari), were enrolled in this multicentric, prospective study. The study was conducted in accordance to the tenets of the Declaration of Helsinki; all study participants signed informed consent. 

### 2.3. Collection of the Conjunctival Samples

From each patient, four conjunctival swabs were collected using the Copan ESwab^®^ collection device (code 480CE, a tube with 1 mL of Amies medium and a flocked swab (FLOQSwabs^®^) (Copan Italia, Brescia, Italy), from each eye at two different time-points. 

### 2.4. Microbiological Determinations

All conjunctival swabs (samples from time T_0_—visit one and T_1_—pre-surgery visit 2), were sent to the Laboratory of Microbiology and Clinical Virology, Monza, Italy, and processed double-blinded for bacterial culture determination on different and appropriate growth media. Trypticase Soy Agar (TSA) Medium, Chocolate Agar Medium, and Laked Horse Blood Agar Medium (Oxoid Thermo Fisher Scientific, Monza, Italy) were inoculated with 100 μL of transport medium for bacterial growth. Each sample was processed in triplicate on each growth medium used. The qualitative microbiological results (type of grown colonies) and quantitative (colony forming units [CFU]/mL) of all types of colonies found were recorded after two days of incubation at 37 °C. 

After the qualitative analysis, which highlighted the different bacterial species present in the medium where the microbial growth took place after the incubation, the count on each plate was performed for each species. Specification at the species level was performed on the various types of isolated colonies, using MALDI-TOF analysis (Vitek^®^ MS, Biomérieux, Italy). Methicillin-resistance phenotype for all isolates of the *Staphylococcus* species (spp.) was also performed.

All strains isolated and identified during the present study, characterized for virulence/pathogenicity factors and antibiotic-resistance profiles, were stored at −80 °C as part of the MicroMiB biorepository, associated member of the Joint Research Unit (JRU) MIRRI-IT (Microbial Resource Research Infrastructure Italian Node), located at the University of Milano-Bicocca, Monza, Italy, for further characterization studies [26].

### 2.5. Statistical Analysis 

Qualitative and quantitative variables were collected in an ad hoc electronic database. Qualitative data were summarized with absolute and relative (percentage) frequencies. Medians and interquartile ranges were used for quantitative variables with a non-parametric distribution. Chi-squared test was used to detect any statistical differences in the comparison of qualitative variables, whereas the Mann-Whitney test was used for the unpaired comparison of the quantitative variables (between treated and untreated eyes). The Wilcoxon test was used to compare total bacterial loads at T_0_ and T_1_. A two-tailed *p*-value less than 0.05 was considered to be statistically significant. The statistical software used was STATA version 16 (StataCorp, College Station, TX, USA).

## 3. Results

### 3.1. Patients’ Descriptive Characteristics

A total of 120 patients, eligible for unilateral cataract surgery, were enrolled in the study but fourteen dropped out due to improper or contaminated conjunctival sampling at T_0_. A total of 106 subjects (64 females, 42 males; median age 74 ± 5 years) were considered valid for the study (Table 1). 

Among them, 39 subjects showed a reduction of total bacterial load while in the remaining, 67 subjects demonstrated no regrowth on the culture media of the pre-existing bacterial flora. 

### 3.2. Primary Outcome Results

#### Microbiological Results from Conjunctival Swabs

From the 424 conjunctival swabs analyzed, Gram-positive bacteria accounted for the most frequently identified microorganisms (85.9%), as compared to Gram-negative bacteria (14.1%). Isolation of fungi occurred sporadically only at T_0_ and at T_1_ in untreated eyes and was not further taken into account in the data analysis. A total of 659 well-identified bacterial species through the MALDI-TOF technique were found to be present on the ocular surface of both eyes from all subjects at T_0_. These overall numbers were reduced to 300 different bacterial species at T_1_ point.

At T_0_, bacterial species were attributable to 3 phyla, belonging to the Firmicutes (64.3%), Actinobacteria (30.7%), and Proteobacteria (5%).

**Figure 1:**
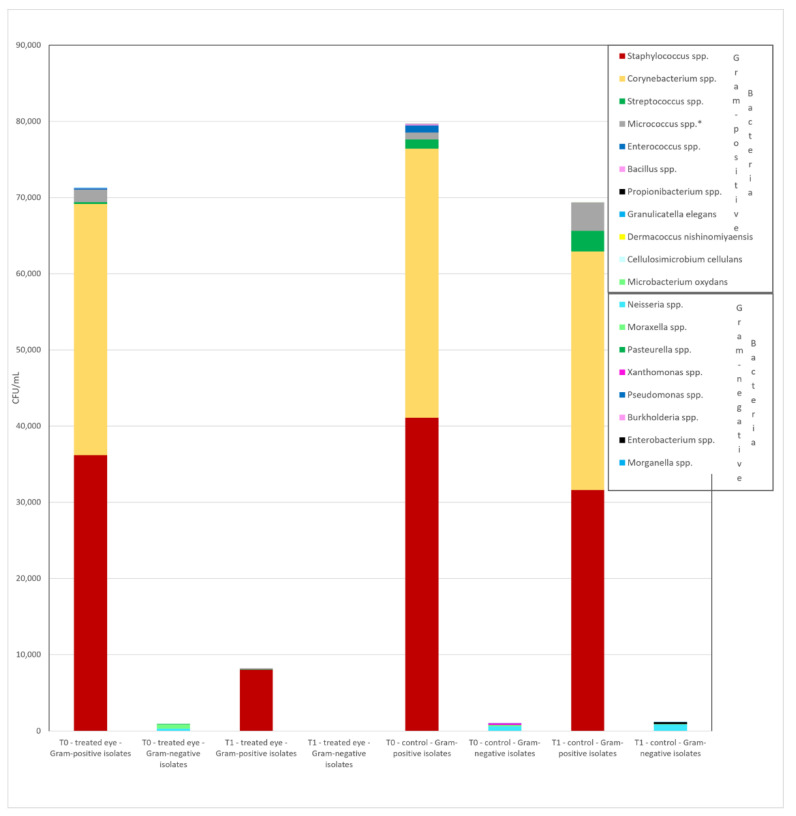
Distribution of Gram-positive and Gram-negative cumulative loads between the treated–untreated eyes at the T_0_ and T_1_ points. spp. = different species; * including *Kocuria* and *Rothia* spp.

**Figure 2:**
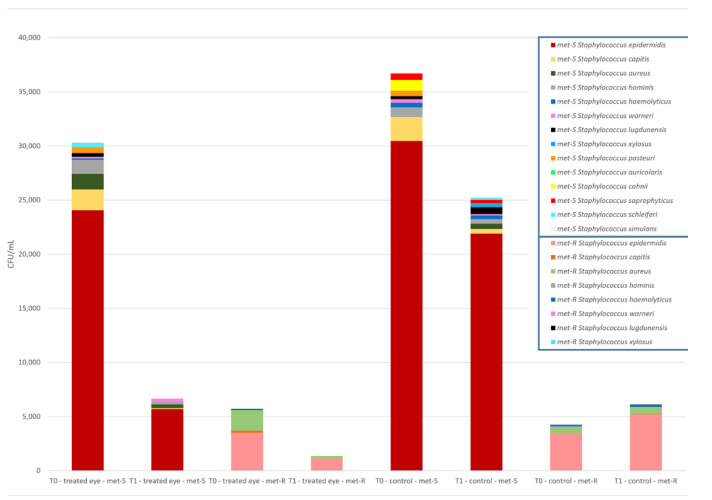
Distribution of methicillin-susceptible and methicillin-resistant *Staphylococcus* spp. cumulative loads between the treated–untreated eyes at T_0_ and T_1_.

The most frequently isolated genera from all conjunctival samples were *Staphylococcus* (59.3%), *Corynebacterium* (21.7%), *Micrococcus* (4.1%), *Kocuria* (2.4%), *Streptococcus* (2.1%), *Neisseria* (2.1%), *Rothia* (1.8%), *Moraxella* (1.1%), and others (5.4%) (Figure 1 and Figure 2).

The most frequent isolated genera found at T_0_ in the eye to be operated were—*Staphylococcus* (58.9%), *Corynebacterium* (21.4%), *Micrococcus* (5.6%), *Kocuria* (2.6%), *Streptococcus* (2.3%), *Neisseria* (3%), *Rothia* (1.3%), *Moraxella* (1.3%), and others (3.6%).

Regarding the most isolated genera at T_0_, 179 staphylococci were found in the eye to be operated, 85 and 94 in the right and left eye, respectively. The most frequently isolated species were *Staphylococcus epidermidis (S. epidermidis)* (57%), *Staphylococcus capitis (S. capitis)* (13.4%), *Staphylococcus aureus (S. aureus)* (7.8%), *Staphylococcus hominis (S. hominis)* (7.3%), *Staphylococcus lugdunensis (S. lugdunensis)* (4.5%), *Staphylococcus pasteuri (S. pasteuri)* (3.9%), *Staphylococcus warneri (S. warneri)* (2.2%), *Staphylococcus haemolyticus (S. haemolyticus)* (1.7%), and others (2.2%).

Methicillin-resistance among all staphylococci was investigated. Thirty-one out of 179 staphylococci were demonstrated to have a methicillin-resistant phenotype (17.4%). The most frequent methicillin-resistant (MR) species was *S. epidermidis* (58.1%), followed by *S. aureus* (16.1%), *S. capitis* (9.7%), *S. haemolyticus* (6.5%), *S. lugdunensis* (3.2%), *S. hominis* (3.2%), and *Staphylococcus xylosus* (3.2%).

Evaluation of bacterial loads at T_0_, associated with all isolated species, demonstrated a range between 8.5 × 10^4^ CFU/mL and 1.1 × 10^2^ CFU/mL. It was interesting to note that polymicrobism was shown in some patients, with up to 6 bacterial species simultaneously present, which lowered the percentage share of individual pathogens but did not cancel the load of some species in favor of others.

At T_1_ point, after the treatment with 0.66% PVI eye drops, in the eye to be operated on, complete eradication of *Corynebacterium* spp., *Streptococcus parasanguinis*, *Moraxella* spp., *Micrococcus* spp., and *Kocuria kristinae* occurred, along with all other Gram-negative bacteria. In the case of *Kocuria rhizophyla* and *Streptococcus mitis/oralis*, a reduction of bacterial positivity of 87.5% and 85.7%, respectively, was observed. 

A significant reduction of total bacterial load after treatment was demonstrated in the treated eye, while the same result was not demonstrated in the untreated eye (Table 2 and Table 3).

At the same time-point in the treated eye, a frequency reduction rate from 179 to 51 for staphylococci was observed (Reduction rate (Rr) of 71.5%). Notably, some species such as *S. lugdunensis*, *S. pasteuri*, *Staphylococcus simulans*, *Staphylococcus cohnii*, *Staphylococcus saprophyticus*, *Staphylococcus schleiferi*, *S. xylosus*, and *Staphylococcus auricularis* were totally eradicated. After treatment with 0.66% PVI eye drops, the most isolated *Staphylococcus* species were *S. epidermidis* (Rr = 64.7%), *S. warneri* (Rr = 75%), *S. capitis* (Rr = 83.3%), *S. hominis* (Rr = 84.6%), *S. aureus* (Rr = 78.6%), and *S. haemolyticus* (Rr = 66.7%). 

Methicillin-resistant staphylococci, belonging to *S. aureus*, *S. capitis*, *S. lugdunensis*, *S. hominis*, *S. haemolyticus*, and *S. xylosus*, isolated at T_1_ in the treated eye, were totally eradicated except for MR *S. epidermidis*, which was isolated in only 5 patients at T_1_ (Rr = 72.2%).

## 4. Discussion

To date, it is known from the literature that postoperative endophthalmitis after cataract surgery has an incidence varying from 0.014% to 0.08%, mainly associated with bacterial species such as *S. epidermidis*, as the major representative of coagulase-negative staphylococci (CNS) (33–77%), *S. aureus* (10–21%), beta-hemolytic streptococci (9–19%), and Gram-negative bacteria (6–22%) [ESCRS Guidelines for Prevention and Treatment of Endophthalmitis Following Cataract Surgery: Data, Dilemmas and Conclusions 2013] [13].

In this clinical study, bacterial species such as staphylococci, *Corynebacterium* spp., streptococci, *Kocuria* spp., and Gram-negatives were demonstrated at T_0_ in participating patients. Methicillin-resistance rates among staphylococcal isolates were found to be 17.4%. In general, methicillin-resistant staphylococci could be resistant, in addition to beta-lactam compounds such as penicillins and cephalosporins, also to tetracyclines, aminoglycosides, and fluoroquinolones. It could be deduced that their removal from the eye surface would greatly reduce the incidence of endophthalmitis supported by non-susceptible bacterial species to most antibiotics routinely used in clinical settings for its treatment. Another consideration related to the management of these ocular infections was that the antimicrobial activity of antibiotics prescribed for topical use could not be evaluated, as to date, there are no updated clinical breakpoints licensed from EUCAST for this use [27], (the official standing committee jointly organized by ESCMID, ECDC, and European national breakpoint committees). 

Moreover, as epidemiological data concerning the bacterial etiology of ocular colonization are scarce and not updated, the MALDI-TOF MS identification of the bacterial strains isolated in this study further contributed to expand the knowledge of potential pathogens found on the eye surface of patients who require cataract surgery. 

In the literature, treatment with PVI used at low concentrations demonstrated microbicidal activity at a concentration in ranges of 0.008% and 0.9% [28]. 

This finding was in keeping with recent studies reporting the in vitro activity of 0.66% PVI against *S. epidermidis*, *S. aureus*, *P. aeruginosa*, and *Candida* species and the efficacy of its treatment in reducing conjunctival bacterial load and risk of needle contamination [24,25].

Moreover, two recent case reports demonstrated the effectiveness of 0.66% PVI in the resolution of signs and symptoms of a corneal ulcer, following treatment failure based on antibiotics [29,30]. 

The following limitations characterized the present study. First, culture-based methods were used to better highlight the bacterial component known for its clinical implications although quantitative sequencing methods could highlight a greater variety of microbial flora. Another limiting aspect was that 0.66% PVI was not compared to other topical antibiotic formulations, although the authors believed that the antiseptic activity and the rationale that foresaw the use of 0.66% PVI eye drops could not be compared to the antibiotic activity and the preventive use of an antibiotic for topical use. Lastly, the present was a non-randomized and not a randomized study. The authors were aware of the fact that the randomized controlled trial (RCT) was universally considered to be the highest level of information in the field of evidence-based medicine.

## 5. Conclusions

In our study, after treatment with IODIM^®^, it was possible to see a reduction of bacterial species present in 100% of the treated patients (no regrowth on culture media or reduction of bacterial load). The most susceptible species to 0.66% PVI treatment were Gram-negatives, followed by *Corynebacterium* spp., *Streptococcus* spp., and *Staphylococcus* spp. In conclusion, the treatment with IODIM^®^ was shown to be effective in reducing the total bacterial load on the eye surface of patients who underwent cataract surgery. These results, further confirmed the findings of other previous clinical studies, suggesting that IODIM^®^ could be a valuable antiseptic adjuvant treatment to the current preoperative prophylaxis of patients undergoing cataract surgery.

## Figures and Tables

**Table 1:** Characteristics of the study population.

Study Group		*n* = 106
Median (IQR) age, years		74 (69–79)
Male, *n* (%)		42 (39.6)
Treated eye, *n* (%)	Right	51 (48.1)
	Left	55 (51.9)

*n* = number of participating subjects.

**Table 2:** Comparison of total bacteria load between T_0_ and T_1_ time-points.

	T_0_	T_1_	*p*-Value
Median (IQR) total bacteria load, treated eye, CFU/mL	325 (140–700)	0 (0–40)	<0.0001
Median (IQR) total bacteria load, untreated eye, CFU/mL	290 (110–930)	250 (90–700)	0.29

IQR = Interquartile range, CFU/mL = Colony Forming Units/mL.

**Table 3:** Comparison of outcomes between the untreated and treated eye.

	Untreated Eye	Treated Eye	*p*-Value
Median (IQR) total bacteria load at T_0_, CFU/mL	290 (110–930)	325 (140–700)	0.54
Median (IQR) total bacteria load at T_1_, CFU/mL	250 (90–700)	0 (0–40)	<0.0001
Median (IQR) total load variation from T_0_ to T_1_	−25 (−250; 170)	−315 (−680; −110)	<0.0001
Bacterial positivity (IQR) at T_1_	96 (90.6)	41 (38.7)	<0.0001

IQR = Interquartile range and CFU/mL = Colony Forming Units/mL.

## Data Availability

Not applicable.
